# Large-scale computational modelling of H5 influenza variants against HA1-neutralising antibodies

**DOI:** 10.1016/j.ebiom.2025.105632

**Published:** 2025-03-17

**Authors:** Colby T. Ford, Shirish Yasa, Khaled Obeid, Rafael Jaimes, Phillip J. Tomezsko, Sayal Guirales-Medrano, Richard Allen White, Daniel Janies

**Affiliations:** a Center for Computational Intelligence to Predict Health and Environmental Risks (CIPHER), University of North Carolina at Charlotte, Charlotte, NC, USA; b Department of Bioinformatics and Genomics, University of North Carolina at Charlotte, Charlotte, NC, USA; c School of Data Science, University of North Carolina at Charlotte, Charlotte, NC, USA; d Tuple LLC, Charlotte, NC, USA; e Massachusetts Institute of Technology, Cambridge, MA, USA; f North Carolina Research Campus (NCRC), Kannapolis, NC, USA

**Keywords:** Avian influenza, Antibodies, Protein modelling, Docking, Zoonosis

## Abstract

**Background:**

The United States Department of Agriculture has recently released reports that show samples collected from 2022 to 2025 of highly pathogenic avian influenza (H5N1) have been detected in mammals and birds. Up to February 2025, the United States Centres for Disease Control and Prevention reports that there have been 67 humans infected with H5N1 since 2024 with 1 death. The broader potential impact on human health remains unclear.

**Methods:**

In this study, we computationally model 1804 protein complexes consisting of various H5 isolates from 1959 to 2024 against 11 haemagglutinin domain 1 (HA1)-neutralising antibodies. This was performed using AI-based protein folding and physics-based simulations of the antibody-antigen interactions. We analysed binding affinity changes over time and across various antibodies using multiple biochemical and biophysical binding metrics.

**Findings:**

This study shows a trend of weakening binding affinity of existing antibodies against H5 isolates over time, indicating that the H5N1 virus is evolving immune escape from our therapeutic and immunological defences. We also found that based on the wide variety of host species and geographic locations in which H5N1 was observed to have been transmitted from birds to mammals, there is not a single central reservoir host species or location associated with H5N1's spread.

**Interpretation:**

These results indicate that the virus has potential to move from epidemic to pandemic status. This study illustrates the value of high-performance computing to rapidly model protein–protein interactions and viral genomic sequence data at-scale for functional insights into medical preparedness.

**Funding:**

No external funding was used in this study.

**Research in context**

**Evidence before this study**

The United States Department of Agriculture has recently released reports that show samples collected from 2022 to 2025 of highly pathogenic avian influenza (H5N1) have been detected in mammals and birds.^1^ Up to February 2025, the United States Centres for Disease Control and Prevention reports that there have been 67 humans infected with H5N1 since 2024 with 1 death.^2^ The broader potential impact on human health remains unclear.

Previous studies have shown that cases of avian influenza transmissions to mammals are increasing in frequency, which is of concern to human health. Since 1997, nearly a thousand H5N1 cases have been reported in humans, with a 52% fatality rate. Previous analyses have indicated specific mutations in the haemagglutinin protein that allow for this “host jumping” between birds and mammals.^3^ There is also existing evidence of recent viral strains with reduced neutralisation by sera.^4^

**Added value of this study**

This study provides a comprehensive look at the mutational space of haemagglutinin of H5N1 influenza and presents computational predictions of the binding between various HA1-neutralising antibodies derived from infected vaccinated patients and humanised mice, and 1804 representative H5 HA1 proteins. These analyses show a weakening trend of existing antibodies. We also confirm that the mutations found in other studies that enable zoonosis also affect binding affinities of the antibodies tested.

Furthermore, through phylogenetic analyses, we quantify the avian-to-mammalian transmissions from 1959 to 2024 and show a persistent circulation of isolates between North America and Europe. Taken together, the continuous transmission of H5N1 from birds to mammals and the increase in strains with immuno-evasive HA in mammals sampled over time suggest that antigenic drift is a source of spillover risk.

**Implications of all the available evidence**

Our findings indicate that the worsening in antibody binding, along with the increase in avian-to-mammalian H5N1 influenza transmissions, are risks to public health.

Through the findings of previous studies along with the predictions reported in this study, we can now monitor specific mutations of interest, quantified by their potential impact on antibody evasion, and inform public health monitoring of circulating isolates in 2024 and beyond. In addition, these findings may help to guide future vaccine and therapeutic development in the fight against H5N1 influenza infections in humans.

## Introduction

Wild aquatic birds (e.g., order Anseriformes) are fundamental hosts of influenza viruses, which are transmitted from domestic birds (e.g., Galliformes) and mammals (orders Artiodactyla, Carnivora, and Primates).^5^^,^^6^ H5N1 has circulated in nature since 1959, following an outbreak in Scotland in chickens.^7^ In 1996, H5N1 influenza largely occurred in Anseriformes and spread to humans and chickens in Hong Kong in 1997.^6^^,^^8^ As a response to H5N1 in chickens and occasional human infection, chickens were culled in Hong Kong in the period 1997 to 2011.^9^ In 2002, the most common hosts of H5N1 were Anseriformes with occasional transmission to Galliformes and humans throughout China and South East Asia.^6^^,^^10^ Since 2003, various lineages of H5N1 have spread throughout China and Hong Kong, South East Asia, Russia, North Africa, the West Bank, Gaza Strip and Israel, Pakistan, Bangladesh, India, Bhutan, Nepal, Europe, Japan and South Korea, using a wide variety of hosts.^11^^,^^12^ Many avian taxa (Charadriiformes, Accipitriformes, Corvidae, Ardeidae, Columbidae, and Passeriformes) as well as primate, carnivore, artiodactyl, and arthropod hosts have been infected with H5N1.^6^ H5N1 infections in humans have been reported by the World Health Organization (WHO) in: Hong Kong 1997–2003, in China and Hong Kong 2003–2014; in Thailand 2003–2007; in Indonesia 2005–2012; in Nigeria in 2007; in Bangladesh 2011–2013; in Azerbaijan, Turkey, Iraq, Myanmar, Pakistan, and Djibouti in 2006–2007; in Egypt 2003–2014; in Lao PDR 2007; in Vietnam 2003–2014; in Cambodia 2003–2014, and in Canada in 2014. The document produced by the WHO has not been updated since.^13^

However, a comprehensive review from 2023^14^ illustrates the recent (2022–2023) spread (on top of the previous spread) of H5N1 in animals as follows:

• **Asia:** Bhutan, Hong Kong, India, Japan, Korea, Nepal, Philippines, Taiwan, and Vietnam.
• **Europe:** Albania, Austria, Belgium, Bosnia and Herzegovina, Bulgaria, Croatia, Czechia, Denmark, Estonia, Finland, France, Germany, Greece, Hungary, Iceland, Ireland, Italy, Latvia, Lithuania, Luxembourg, Moldova, Montenegro, Netherlands, North Macedonia, Norway, Poland, Portugal, Romania, Russia, Serbia, Slovakia, Slovenia, Spain, Sweden, Switzerland, and the United Kingdom.
• **The Middle East:** Israel and Turkey.
• **Africa:** Algeria, Gambia, Nigeria, Reunion, Senegal, and South Africa.
• **North and South America:** Bolivia, Brazil, Canada, Bolivia, Chile, Colombia, Ecuador, Guatemala, Honduras, Mexico, Panama, Peru, Venezuela, and the United States.

In 2024, H5N1 has been found in animals in Antarctica.^15^ Also, in March 2024, an outbreak of H5N1 was reported among several herds of U.S. dairy cattle. H5N1 also caused fatal infections among cats, infection in poultry, and four reported infections in dairy workers.^16^, ^17^, ^18^ From 1997 to late April 2024, 909 human H5N1 cases were reported, with 52% of cases being fatal.^19^

Continued transmission of avian strains of H5N1 to livestock and humans may lead to subsequent human-to-human transmission which pose serious risks to public health worldwide. As the human–animal interface increases due to shrinking natural habitats, deforestation, and increased demand for animal products; animal-human disease transmission is becoming more common.^20^

Current human seasonal influenza vaccines do not confer immunity against H5N1 influenza or other animal influenza A viruses.^21^ Moreover, recent studies have shown there is little existing immunity to H5N1 in the USA.^22^ Such immunity may exist elsewhere in the world due to previous infection or immunisation.

Thus, it is of great public health interest to discover rapidly develop molecular insight into the effects of mutations of H5N1 on existing human immunity.^23^ In this study, we present the results of a large computational corpus of molecular docking experiments between various H5 isolates against existing HA1-neutralising antibodies and show changes over time.

## Methods

**Fig. 1:**
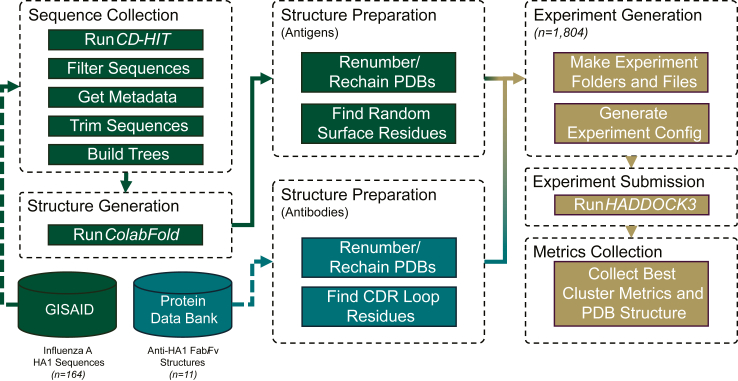
Workflow diagram of the data procurement, data preparation, and analysis steps.

This study closely follows the published methods in Tomezsko and Ford et al. (2024) and other protein modelling studies by this team.^24^, ^25^, ^26^, ^27^, ^28^ The specific workflow for this study is depicted in Fig. 1.

### HA1 sequence collection

18,693 influenza A H5 sequences were downloaded from the GISAID EpiFlu database^29^^,^^30^ on June 17, 2024. Isolate metadata, including date of viral isolation, country of origin, and host information were also collected. We then derived taxonomic classifications from the provided host metadata along with continent of origin from the country information.

These amino acid sequences were clustered using CD-HIT v4.8.1.^31^^,^^32^ Ranging in size from 11 to 576 amino acids, the process resulted in 250 clusters, based on 97% identity. A representative sequence from each of these clusters was selected, which was then output in a FASTA file.

These representative sequences were then aligned to each other with MUSCLE v3.8.425^33^ using default settings. Then, sequences that were of low quality, incomplete, or did not contain the desired haemagglutinin domain 1 (HA1) region were removed. This resulted in 164 amino acid sequences that were used in the subsequent steps. These sequences were trimmed to the HA1 receptor binding domain (approximately residues 111–269, depending on the presence of indels).^34^

Also, clusters consisting of only lab derived isolates (*n* = 3) were analysed, but have been left out of the reported statistics and visualisations.

### Structure prediction

Structures for each of the 164 HA1 sequences were predicted using ColabFold v1.5.5,^35^ a protein folding framework that uses AlphaFold2^36^ accelerated with MMseqs2,^37^ with default settings. The side chains of these predicted structures were relaxed using the OpenMM/Amber method.^38^ The .PDB file output of the top structure (i.e., the one with the highest predicted local distance difference test (pLDDT) confidence) was selected and used for subsequent analyses. pLDDT is an estimate of local confidence, scaled from [0, 100], where higher scores indicate higher confidence in the protein conformation.

### Neutralising antibody collection

Existing structures for 11 HA1-neutralising antibodies were collected by a database search through Thera-SAbDab^39^ and the Protein Data Bank.^40^ Each of these 11 antibody structures have an epitope on the target HA1 domain of the haemagglutinin protein, though not all share the same epitope. These antibodies and their respective PDB IDs are listed in Table 1.

**Table 1:** HA1-neutralising antibodies.

Antibody ID	PDB ID	Year (Reference)	Clade	H/L Chain Subgroups	Source Information
100F4	5dur	2015^41^	2.3.4.4	II/I	Human Memory B-Cell, Recovered from H5N1 Infection
12H5	7fah	2022^42^	2.3.4.4	I/IV	Mouse, Immunised with three H1N1 strains, Humanised
13D4	6a0z	2018^43^	2.3.2.1c	I/I	Mouse, Immunised with five H5N1 strains, Humanised
3C11	6iuv	2019^44^	2.3.4.4	I/II	Human Memory B-cell, Infected by H5N1 viruses
65C6	5dum	2015^41^	2.3.4.4	I/III	Human, Infected by H5N1 viruses
AVFluIgG01	6iut	2019^44^	2.3.4.4	II/I	Human, Infected by H5N1 viruses
AVFluIgG03	5dup	2015^41^	2.3.4.4	III/I	Human, Infected by H5N1 viruses
FLD194	5a3i	2015^45^	2.3.4.4	II/I	Human Memory B-cell, Recovered from H5N1 infection
FLD21.140	6a67	2018^46^	2.3.4.4	?/I	Human, Recovered from H5N1 Infection
H5M9	4mhh	2013^47^	2.3.2.1	I/IV	Mouse, Immunised with H5N1, Humanised
H5.3	4xrc	2015^48^	2.3.4.4	II/?	Human, Immunised with one H5N1 strain

### Docking analyses

Using HADDOCK3, a computational framework for the integrative modelling of biomolecular complexes,^49^ each antibody was docked to each antigen across the dataset. Given the 11 antibody structures and 164 HA1 structures, this resulted in 1804 docking experiments to be performed.

#### Experiment generation

HADDOCK3 inputs for each experiment were generated programmatically, defining the antibody and antigen. PDB file inputs on which to dock. Other experiment files were also copied or created programmatically including the scripts to run the docking process and to generate other configuration files.

HADDOCK3 requires the definition of active and inactive residue restraints (AIRs) to help guide the protein docking process. To avoid biasing the docking placement of the antibody on the HA1 antigens, a random subset of surface residues was selected as “active” and these were then included in the AIR file on which to dock. For the antibody structures, residues in the CDR loops were detected using ANARCI,^50^ a Python package for numbering antibody sequences.

Lastly, HADDOCK3 configuration files were generated programmatically, which define the input. PDB files, the output directory, and the steps of the docking process. The logic for this programmatic generation of HADDOCK3 configuration files is available in the GitHub repository listing in the Supplementary materials for this manuscript.

#### Docking process

HADDOCK3 provides a configurable interface for defining the individual steps of the docking process, including the rigid-body docking, flexible refinement, and solvent-based refinement, along with any desired clustering and filtering steps.

For this study, we customised the published HADDOCK3 protocol for antibody-antigen modelling^51^ to focus on generating the singular best cluster of docking results for each experiment and reducing excess work by the docking process. The specific steps of our HADDOCK3 configuration that was used for the experiments are shown in Table 2.

**Table 2:** Descriptions of the steps of the HADDOCK3 docking configuration.

Step	Description	# Models
1 Topology Modelling	Creates CNS all-atom topology	–
2 Rigid Body Modelling *n* = 200	Rigid body energy minimisation with CNS (*it0*). Samples *n* models.	200
3 Cluster with FCC	Calculates the contacts between chains and then calculates the FCC matrix for each model. Clusters the models based on the calculated contacts.	200
4 Select Top Clusters *n* = 5, *m* = 10	Selects top *n* clusters of *m* models based on HADDOCK score	50
5 Flexible Refinement	Semi-flexible refinement using a simulated annealing protocol. Uses molecular dynamics simulations in torsion angle space (*it1*)	50
6 Cluster with FCC	Calculates the contacts between chains and then calculates the FCC matrix for each model. Clusters the models based on the calculated contacts.	50
7 Select Top Cluster *n* = 1, *m* = 10	Selects top *n* clusters of *m* models based on HADDOCK score	10
8 Solvent Refinement	Refinement by a short molecular dynamics simulation in explicit solvent (*itw*)	10
9 Molecular Dynamics Scoring	Performs a short molecular dynamics simulation on the input models and scores them.	10
10 Cluster with FCC	Calculates the contacts between chains and then calculates the FCC matrix for each model. Clusters the models based on the calculated contacts.	∼10
11 Calculate CAPRI Metrics	Calculates i-RMSD, I-RMSD, Fnat, DockQ of the top scoring models.	∼10

The HADDOCK3 system outputs multiple metrics for the predicted binding affinities and an output set of PDB files containing the antibody docked against the HA1 antigen. Some main metrics include:

• Van der Waals intermolecular energy (*vdw*) in kcal/mol
• Electrostatic intermolecular energy (*elec*) in kcal/mol
• Desolvation energy (*desolv*) in kcal/mol
• Restraints violation energy (*air*) in arbitrary units
• Buried surface area (*bsa*) in Å^2^
• Total energy (*total*): 1.0*vdw* + 1.0*elec* in kcal/mol
• HADDOCK score: 1.0*vdw* + 0.2*elec* + 1.0*desolv* + 0.1*air*

Note that the HADDOCK Score is a conglomerate metric used to assess the best complexes (or best cluster of complexes) that get promoted through the various refinement iterations in the pipeline.

#### Computational scalability

For this study, we used a Docker containerised version of HADDOCK3 (Container GitHub Repository: https://github.com/colbyford/HADDOCKer; Docker Hub Images: https://hub.docker.com/r/cford38/haddock), which contains all of the software dependencies to allow HADDOCK3 to run more readily in a high-performance computing (HPC) environment.

HADDOCK3 was run in a Singularity container on the UNC Charlotte Orion HPC cluster on 14 nodes, each with dual 18-core Intel Xeon Gold 6154 3.00 GHz CPUs (36 cores per node). The average elapsed real time of the experiments was 14.63 min. The entire set of 1804 experiments was completed in under 2 days.

#### Post processing

Once all experiments were completed, the metrics for each experiment were either retrieved from the CAPRI evaluation outputs (if the FCC clustering algorithm^52^ reached convergence) or from the REMARK entries of the best cluster's.PDB files. These metrics were organised in a single aggregate table, representing each experiment's best cluster metrics, for subsequent visualisation and statistical analyses. The full table of experiment results is available in the Supplementary Data.

### Phylogenetic and protein structure analyses

**Fig. 2:**
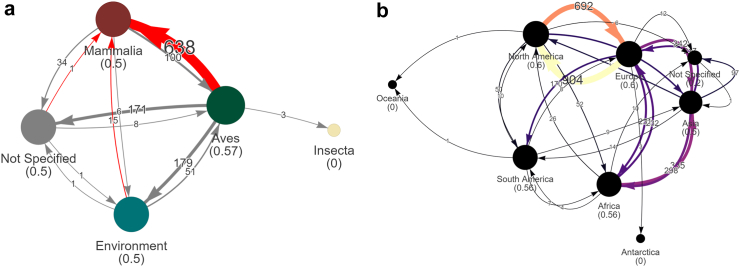
Transmission networks, generated by StrainHub, showing transmissions between (a) hosts and (b) continents. Node sizes and their values in parentheses represent the source-hub ratio of that class or location. A source-hub ratio of 1 indicates that the state is always the source of the transmission. The edge widths and numerical labels annotated on the edges of the graphs represent the number of transmissions as seen across the phylogenetic tree's branches as measured in changes in metadata states. In Subfigure (a), the colours of the nodes correspond to the host category used in other figures and the red-coloured edges annotate transmissions to/from mammals. In Subfigure (b), the edges are coloured from black to orange, indicating a low-to-high number of transmissions, respectively.

For phylogenetic analyses, laboratory derived isolates were filtered out (*n* = 178, shown in Table 3), resulting in a set of 18,515 isolates. An alignment of 18,515 HA sequences was generated using MAFFT v7.471^53^ under default settings. Next, a phylogenetic tree search was performed using this alignment with TNT v1.6^54^ using the commands: xmult = level 1 rep 1000. The heuristically best scoring tree was used for downstream analyses. StrainHub v2.0.0^55^ was used to generate transmission networks of the phylogenetic tree by host class and continent in Fig. 2.

**Table 3:** Taxonomic breakdown of the isolates used in this study.

Class	Order	Isolates	% of Total
Aves	Galliformes	5844	31.26%
	Anseriformes	4469	23.91%
	Other orders	934	5.00%
	Not specified	5287	28.28%
	*Aves total*	*16,534*	*88.45%*
Mammalia	Primates (Humans)	666	3.56%
	Carnivora	191	1.02%
	Artiodactyla	57	0.30%
	Other orders	6	0.03%
	Not specified	360	1.93%
	*Mammalia total*	*1280*	*6.85%*
Insecta	*Insecta total*	*3*	*0.02%*
Other	Laboratory derived	178	0.95%
	Other/Environmental	698	3.73%
	*Other total*	*876*	*4.69%*
**Grand Total**		**18,693**	**100%**

Visualisations and analyses of the protein complexes were generated using PyMOL v2.4.1^56^ and BioPandas v0.4.1.^57^

### Statistics

Nonparametric statistical analyses were performed to evaluate the differences in antibody binding affinity. This includes Spearman correlations for evaluating significant affinity changes over time and Wilcoxon Rank Sum tests for evaluating affinity changes by mutation.

Statistical analyses were performed using R v4.3.4^58^ and plots were generated using ggplot2^59^ and ggpubr.^60^ Any statistical significance reported in this study is based on a p-value threshold of *α* < 0.05.

#### Graph-based interface residue assessment

Graph-based interface residue assessment function (GIRAF) was employed to evaluate the evolution of the binding pocket with each antibody as previously described.^61^ The outgroup was first selected as sequence EPI242227, and a graph was computed based on the interface residues with each antibody to generate reference complexes. Subsequent graphs were then computed for each strain sequence and antibody pair. The graph edit distance (GED) was computed as the number of edits to the strain:antibody complex from the reference outgroup:antibody complex. Substitutions, deletions, and additions were all equally weighted as a value of “1”.

### Ethics

No human or animal samples were used in this study. This study was conducted in accordance with the data usage guidelines of GISAID and the research ethics policies of the University of North Carolina at Charlotte.

### Role of funders

No external funders were used in this study and thus played no role in the study design, data collection, data analyses, interpretation, or writing of the manuscript.

## Results

From the study set of 18,693 H5 isolates, we show a breakdown of hosts similar to what has been reported in previous studies, indicating the representative nature of our curated dataset.^5^^,^^6^ As shown in Table 3, approximately 88% of the isolates are from birds (class Aves). Also, note that all 666 isolates categorised as Primates were collected from humans.

As shown in Supplementary Fig. S2, the proportion of H5 isolates collected from various continents has changed over time. To date, isolates collected in 2024 are predominately from Europe.

### Sequence analyses

The clustering of the 18,693 HA1 sequences resulted in 250 distinct clusters at ≥97% identity. Further organisation of the representative sequences of each cluster, shown in Supplementary Fig. S3, indicates a continuous distribution of antibody binding performance.

Of note, antibodies 12H5, 3C11, 65C6, AVFlulgG01, and H5M9 show the best binding performance overall across the set of metrics, though there are exceptions of poor binding (e.g., 12H5’s interaction with EPI893474 has a poor Van der Waals energy of −39.93).

#### Phylogenetic and transmission network analyses

Specifically, the most common pattern is avian-to-mammalian transmission, of which there are over 600 events across the tree (see Fig. 2a). There are also frequent transmission events between many continents. Bidirectional transmissions between Europe and North America are very frequent as shown by the thick orange and yellow lines in Fig. 2b.

### Worsening binding affinity

**Fig. 3:**
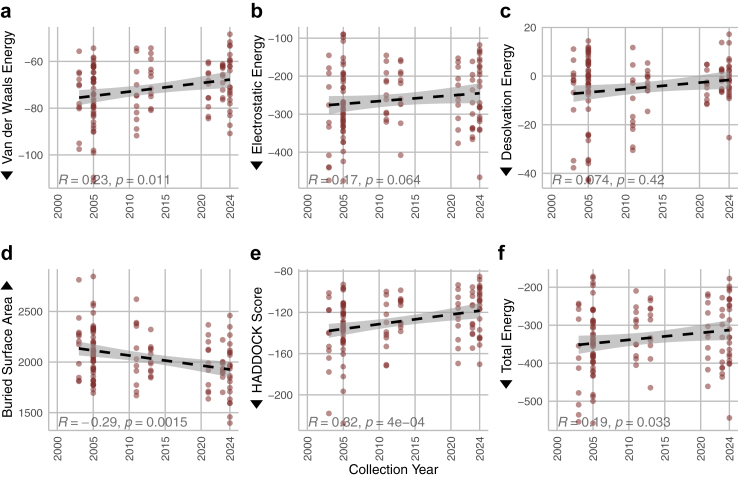
Antibody binding performance metrics over time for isolates collected from humans. Subfigures represent separate binding affinity metrics: a) Van der Waals energy, b) electrostatic energy, c) desolvation energy, d) buried surface area, e) HADDOCK score, and f) total energy. Statistics shown are Spearman correlations. Overall, these plots show a worsening trend in most antibody binding metrics of the human samples. Arrows indicate the “better”, i.e., stronger, direction for binding affinity (*n* = 121).

When considering a past-to-present trend in viral collection date, there are significant correlations that show a worsening in antibody binding affinity in isolates collected from humans. See Fig. 3. This worsening in binding affinity is not specific to particular antibodies. Though the antibodies’ performances are from independent distributions, as shown by the Kruskal–Wallis test in Supplementary Fig. S4, the overall trend indicates that more recent isolates have mutated to better evade existing antibodies (antibodies that were previously elicited by vaccination or infection or developed for therapeutic usage).

**Fig. 4:**
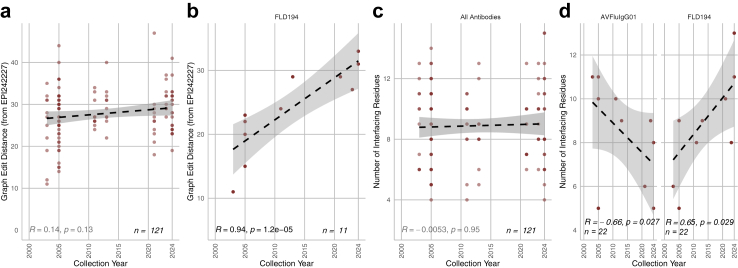
Graph-based analysis results showing the correlation of the graph edit distance and interfacing residue counts against collection year. Subfigures a and c show the results of all antibodies. Overall, graph edit distance did not change significantly over time. Subfigures b and d show Spearman correlations for specific antibodies of interest. Graph edit distance increased significantly in humans for FLD194. The number of interfacing residues increased in FLD194 in humans.

As shown in Fig. 4a, there is no overall trend in the graph edit distance of the isolates over time. In other words, when comparing to the 1959 isolate, EPI242227, interactions are not necessarily more or less abundant in more recent isolates than older ones overall. However, for some antibodies, there is a significant correlation, such as in FLD194 in humans (shown in Fig. 4b).

As shown in Fig. 4c, we see a statistically significant decrease of interfacing residues over time in antibodies AVFluIgF01 against human isolates. Conversely, we see a statistically significant increase in interfacing residues in FLD194 against human residues in Fig. 4d.

These results indicate an overall worsening in antibody affinity to more recent H5N1 isolates, which poses a risk to public health in that the virus may evade existing antibodies and risk the development of severe sickness in humans.

#### Mutational effects

As indicated in Shi et al. (2014), there are various sites in the HA1 receptor binding domain that enable infection of mammals when mutated. Our results show several statistically significant differences in the binding affinity of antibodies given polymorphism at sites that allow mammalian infection.

Of note, N158S, T160 A/S/V, E190N, and G225R all result in weakened antibody binding affinity across multiple metrics. Conversely, T160K and G228S increase binding affinity in some metrics. Significant changes based in Van der Waals energies and HADDOCK Scores are shown in Fig. 6 and all other metrics are shown in Supplementary Fig. S5.

**Fig. 5:**
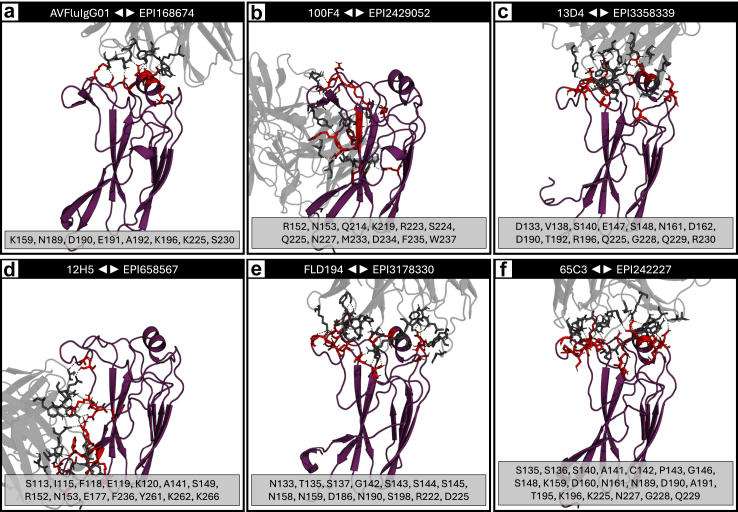
Example interface renderings showing the diversity in epitopes, residues, and binding affinity. The grey structure is the Fab portion of the docked antibody, and the purple structure is the HA1 antigen with side chain sticks designating the polar contacts between them. The list below each subfigure contains the interfacing residues on the antigen chain. Subfigures show various interface examples of interest, including: (a) the worst binding interaction across the set of experiments with antibody AVFluIg01 and H5 isolate EPI168674; (b) the best binding interaction across the set of experiments with antibody 100F4 and H5 isolate EPI2429052; (c) antibody 13D4 and the H5 isolate EPI3358339 that resulted in the death of a man in Mexico in 2024; (d) improved binding affinity of antibody 12H5 and H5 isolate EPI658567 not due to G225R; (e) the poor binding affinity of antibody FLD194 and isolate EPI3178330 due to the E190N mutation; and (f) antibody 65C3 with reference H5 isolate EPI242227 from 1959.

In Fig. 5, subfigures a and b represent the worst and best binding structures across the experimental results, isolates EPI168674 and EPI2429052, respectively. Though the epitope is different between isolates, note the variation in quantity of polar contacts within the respective complexes. Fig. 5d is an example of modest binding affinity between an isolate EPI658567 and antibody 12H5. However, this improved binding affinity compared to other isolates is not due to the G225R mutation as this residue is not in the epitope of the antigen.

**Fig. 6:**
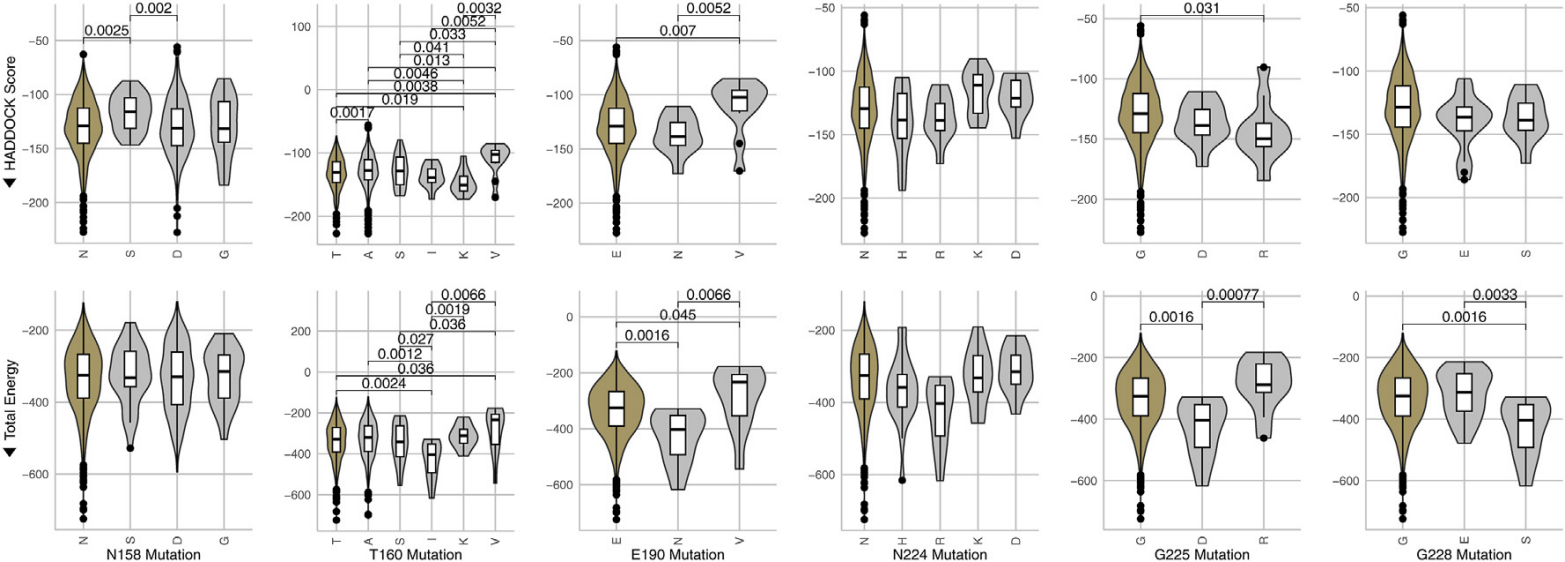
The distribution of Van der Waals energies and HADDOCK score docking metrics broken out by antigen mutations. The first amino acid shown on the left of each plot in gold represents the reference residue at that position as described in Shi et al. (2014). Statistical comparisons shown are significant Wilcoxon Rank Sum test p-values at the *α* < 0.05 level. Arrows indicate the “better” direction, i.e., stronger binding affinity (*n* = 1771).

Fig. 5e shows the poor binding affinity of isolate EPI3178330 with antibody FLD194 due to the E190N mutation. However, this residue forms a polar contact with the antibody CDR loop. The mutation from glutamic acid (E), a negatively charged side chain, to asparagine (N), a polar uncharged side chain.

Isolate EPI242227 is the oldest isolate in the dataset, collected in 1959. Note that this isolate contains the N158D mutation and its interaction with various antibodies results in a wide range of Van der Waals energies from −52.81 (weakest) to −92.84 (strongest, with antibody 65C3 as shown in Fig. 5f).

#### Interfacing residue prevalence

**Fig. 7:**
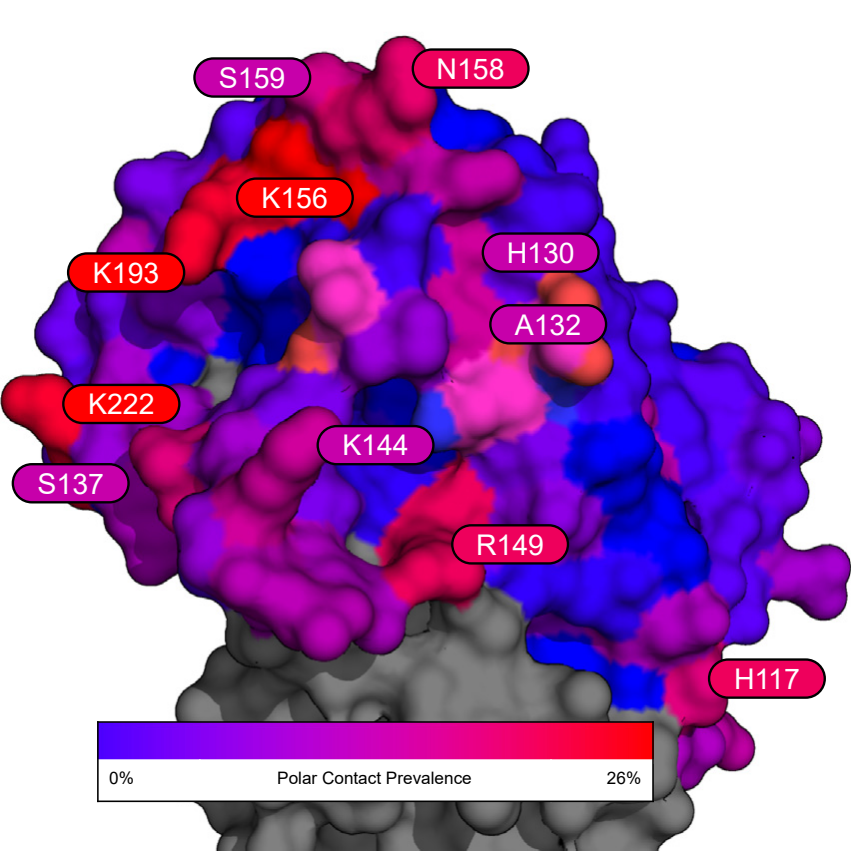
Surface rendering of the HA1 globular head domain (reference PBD 2FK0) showing the prevalence of each residue to form polar contacts (within 3Å of antibody residues) across the experiments in this study. Annotated residues are those with ≥16% prevalence.

An analysis of the interfacing residues in the best complexes from all 1804 experiments shows patterns in particular residues forming polar contacts with the antibodies tested. Residues 156, 193, 222 are often interfaced (≥25% prevalence) in the antigen epitope. These are shown in red in Fig. 7.

Many of the frequently interfaced residues shown in this study closely neighbour antigenic and receptor binding sites reported in Sriwilaijaroen and Suzuki (2012) Fig. 2. Residues 130, 132, 158, and 159 are part of two glycosylation sites that, in 2012, were only found in seasonal H1 viruses.^34^

## Discussion

### Relation to prior studies

This *in silico* analysis yields the trend of a reduction in binding affinity for neutralising antibodies against H5N1-designated influenza isolates. This reduction in binding affinity reinforces previous studies that evolution has occurred to yield HA proteins that are elusive of antibody neutralisation.^62^^,^^63^ The trend observed is consistent with empirical studies and strengthens the *in silico* approach taken within this study. Current biosurveillance efforts focus on critical mutations that have been shown to increase virulence or transmission risk, such as those included in the influenza risk assessment framework (IRAF).^64^ This work suggests that the current arsenal of broadly neutralising antibodies against H5N1 is becoming increasingly insufficient as H5N1 evolves, indicating a need for more studies to identify effective antibodies.

### Diversity of H5N1 interactions

As shown in Supplementary Fig. S3, high disparities in antibody binding affinity exist between different sequences designated as H5N1. For example, in Supplementary Fig. S3, sequence EPI597824 has a large disparity in Van der Waals energy between antibody 3C11 and AvFluIgG03. In addition, Supplementary Fig. S5 demonstrates the effect of a single amino acid mutation on docking metrics. One change in an amino acid residue can lead to statistically significant differences in antibody binding affinity. These disparities highlight the need to continue to elucidate how differences in amino acid sequences alter binding affinity to various antibodies. Categorising H5N1 influenza outside of the primary amino acid sequence but on functional binding analyses may yield more effective treatments in the future.

### Zoonosis analysis

In Fig. 2a, a notable transition pattern is observed from avian species (class Aves) to mammals (class Mammalia), likely attributable to a mutagenic drift. Recent empirical studies have investigated the mutational dynamics of H5N1, revealing changes in the haemagglutinin (HA) protein. While H5N1 influenza prefers binding to the *α*2–3 sialic receptors in birds, our study demonstrated a binding affinity of H5N1 to *α*2–6 sialic acid receptors, predominant in mammals, at almost equal proportion.^4^ These authors also show that mutations that decrease neutralisation by sera from mice and ferrets immunised with the vaccine candidate reference strain A/American Wigeon/South Carolina/USDA-000345-001/2021 exist in some of the most recently collected mammalian samples, the dairy cow outbreak starting in April 2024.^4^ Concurrently, we show here that mutations accumulated over time in the HA protein will confer reduced neutralisation by antibodies more broadly than the current clade 2.3.4.4b H5N1 outbreak.

This *in silico* study aligns with these findings, indicating a progressive decrease in H5N1's binding affinity to antibodies in our isolates over time. As illustrated in Fig. 3, there is a marked decline in affinity for human isolates. As the virus diversifies in the avian populations, the potential pool of strains with zoonotic potential to infect mammals increases. The phylogenetic and transmission analysis show much more frequent transmission from avian populations to mammalian populations. This result indicates that much of the evolution is occurring in birds. This suggests an evolutionary trajectory in birds for H5N1 towards increased infection in mammalian hosts with a concomitant immune evasion of the virus in mammals.

### Isolate EPI3358339

EPI3358339, a H5N2 subtype isolate, was added to this study as it is from the recent human infection of H5N2 avian influenza seen in Mexico. Unfortunately, this strain was found in a person from Mexico who died of complications due to the infection. However, it is not yet known if the zoonotic “jump” seen in this isolate is cause for concern, or if the individual had other comorbidities that played a role in their death. It is also not known if this case is related to recent poultry outbreaks in the area.

The experimental docking conformation between antibodies and this antigen (antibody 65C3 shown in Fig. 5f) are predicted to have a relatively strong binding affinity (e.g., Van der Waals energy: [−51.96, −95.45]) across all the tested antibodies.

Thus, our experiments using this isolate's HA do not indicate that this isolate is highly mutated, though some mutations may have reduced the Van der Waals and electrostatic energies of the interaction with this individual's existing antibodies, if any.

### Structural analysis

When comparing the predicted docking outputs to empirical structures, such as those listed in Table 1, we see similar binding epitopes in the predicted docking complexes versus the empirical structures.^41^, ^42^, ^43^, ^44^, ^45^, ^46^, ^47^, ^48^

Furthermore, the binding conformations seen in the empirical structures often mimic the predicted complexes in the experiments in this study, though various mutations affect the binding angle, polar contacts, electrostatics, and overall affinity. These results support the confidence in the predictive accuracy of the *in silico* experiments given their similarity to empirically derived structures. However, empirical studies are still needed to validate specific complexes.

### Motifs of interest and future research

H5N1 exists as an endemic in avian populations. This endemic creates a pool containing vast numbers of host species in which the RNA virus evolves rapidly. It is an endemic that is difficult to diminish due to the nature of the HA protein's host receptors lacking homogeneity. Serological immunity from vaccination or prior infection in avian hosts may have yielded selective pressures in the evolution of specific mechanisms of entry for H5N1.^65^ Subsequently, high infection rates lead to new mechanisms of entry. Over time, serological immunity from original vaccination and/or infection is reduced, and the cycle of influenza transmission continues.

Highly conserved portions of HA are of high interest. The three primary conserved elements of the receptor-binding site (RBS) on the HA1 subunit are the 130 and 220-loops and the 190-helix.^66^, ^67^, ^68^ As shown in Fig. 5, Fig. 7, the antibodies docked to conserved motifs on the studied H5N1 strains, further supporting the empirical literature that initially identified their neutralising capability.

More recent development of multivalent mRNA-based vaccines has been successful in H5 influenza A clade 2.3.4.4b (from which there are 15 sequences used in the structural aspects of this study).^69^ The selection of high-quality sequences that elicit strong antibody responses is a complex process in mRNA vaccine development. However, *in silico* modelling, as presented in this study, reduces the wet laboratory workload to evaluate candidate sequences from which vaccines can be developed.

In addition, our broad analyses of various antibodies versus strains of interest may guide future therapeutic antibody development. Antibodies tested within this study, particularly those with high affinity to studied strains that may bear high homology to those that will arise in the future, can be used as a basis for future pharmaceutical development.

Computational modelling of immunoprotein interactions as shown in this study and previous works^24^, ^25^, ^26^, ^27^, ^28^, ^61^ have proven to be highly effective in the prompt prediction and understanding of the health impacts of pathogen variants. For H5 influenza, this study, along with recent publications,^4^^,^^18^ show an overall trend of worsening antibody binding and depicts the recent increase in avian-to-mammalian transmissions due to various mutations. This suggests that there is an impending danger to human health for highly pathogenic strains of H5 influenza that can infect avian and mammalian livestock and jump to humans.

More broadly, these results indicate that the virus has potential to move from epidemic to pandemic status. “Pandemic” here refers to the geographic spread of a virus, which H5N1 has already achieved, but these results more specifically assert that the worsening trend of the antibody performance along with the already present animal pandemic is a cause for concern for an eventual human pandemic.

### Limitations

While this study includes over 18,000 H5 influenza A isolates and evaluates the binding affinity of 11 HA1-neutralising antibodies with each, there are limitations into the computational approach taken. While we feel the predicted binding sites and affinities are accurate, future empirical studies should be performed to elucidate particular findings of interest. Specifically, more recent avian influenza isolates that pose specific risks to human health due to their ability to jump from avian to mammalian hosts.

## Contributors

Authors SY, KO, and SGM performed data curation of the H5 antigen sequences from GISAID and antibody structures from Protein Data Bank. SY performed the formal analysis of the structure prediction of the antigens. Author RAW performed the clustering analysis. Authors KO and CTF curated the metadata of the H5 sequences. Author DJ performed the phylogenetic analyses. Authors SY and CTF performed the protein structure analyses. Author PJT performed the multiple sequence alignment. Author RJ3 performed the graph-edit distance analysis. Author CTF performed the docking experiments and statistical analyses and generated all visualisation figures. SY and CTF accessed and verified the underlying data. All authors wrote the original draft of the manuscript. Author CF edited the manuscript revision. All authors read and approved the final version of the manuscript.

## Data sharing statement

All code, data, results, and additional analyses are openly available on GitHub at: https://github.com/colbyford/Influenza_H5-Antibody_Predictions. These data include all sequences and folded structures for the isolates and antibodies used in this study, analysis scripts, and docking metrics.

## Declaration of interests

Author CTF is the owner of Tuple, LLC, a biotechnology consulting firm. The remaining authors declare that the research was conducted in the absence of any commercial or financial relationships that could be construed as a potential conflict of interest. Author DJ is the director of the UNC Charlotte Center for Computational Intelligence to Predict Health and Environmental Risks (CIPHER), which is funded by an Ignite grant from the UNC Charlotte Division of Research.

## Appendix A Supplementary data

Barchart_class_by_year

Barchart_continent_by_year

Dendro_vdw_grid

Boxplots_by_antibody_grid

Boxplots_by_mutation_metric_grid_vdw
