# Supplementary figures and images for: Large-scale computational modelling of H5 influenza variants against HA1-neutralising antibodies
Source: eBioMedicine. 2025 Mar 17;114:105632. doi: 10.1016/j.ebiom.2025.105632 (PMC11960665; doi:10.1016/j.ebiom.2025.105632)

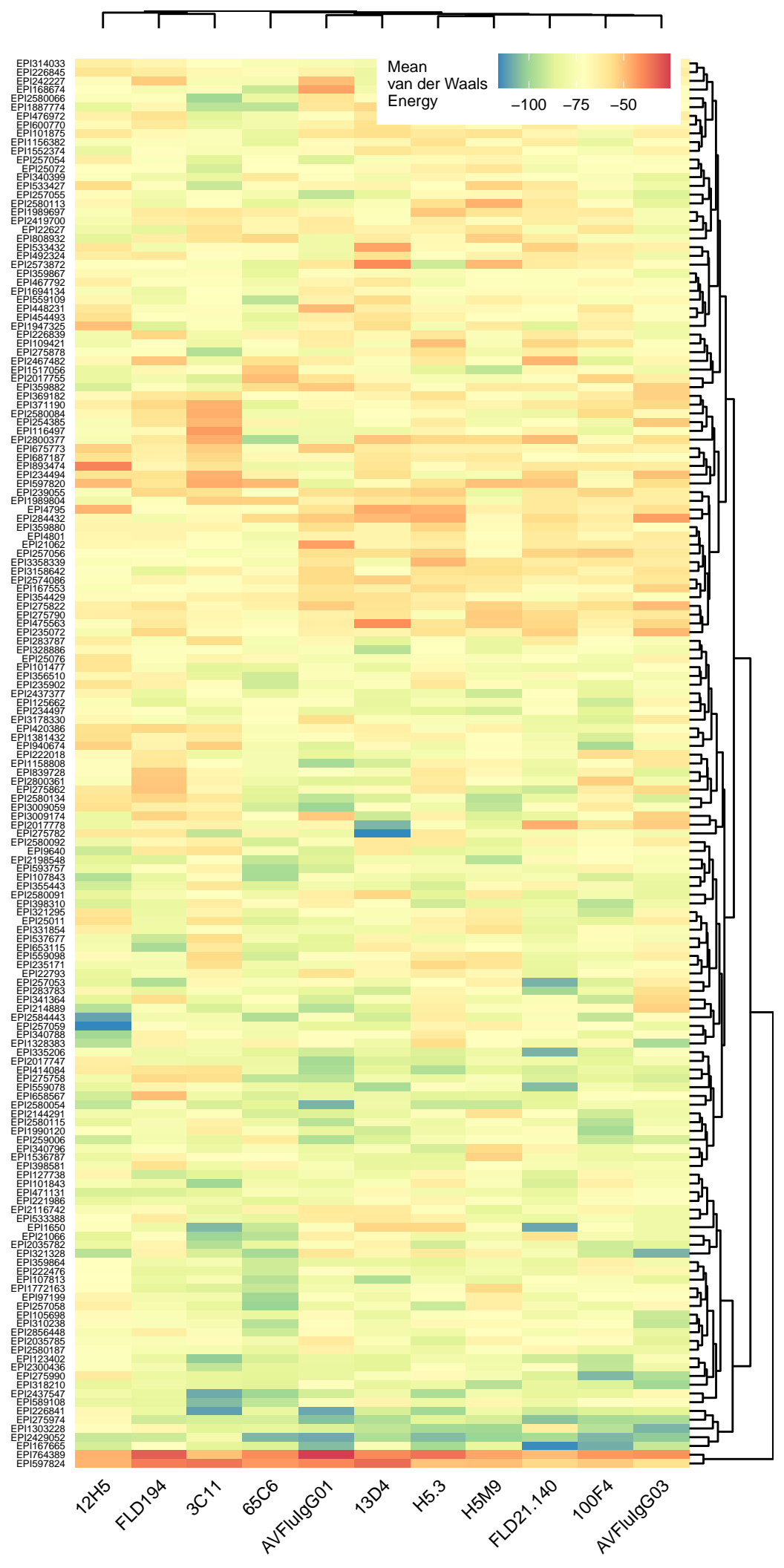

Supplement: Dendro_vdw_grid [file mmc3.pdf]

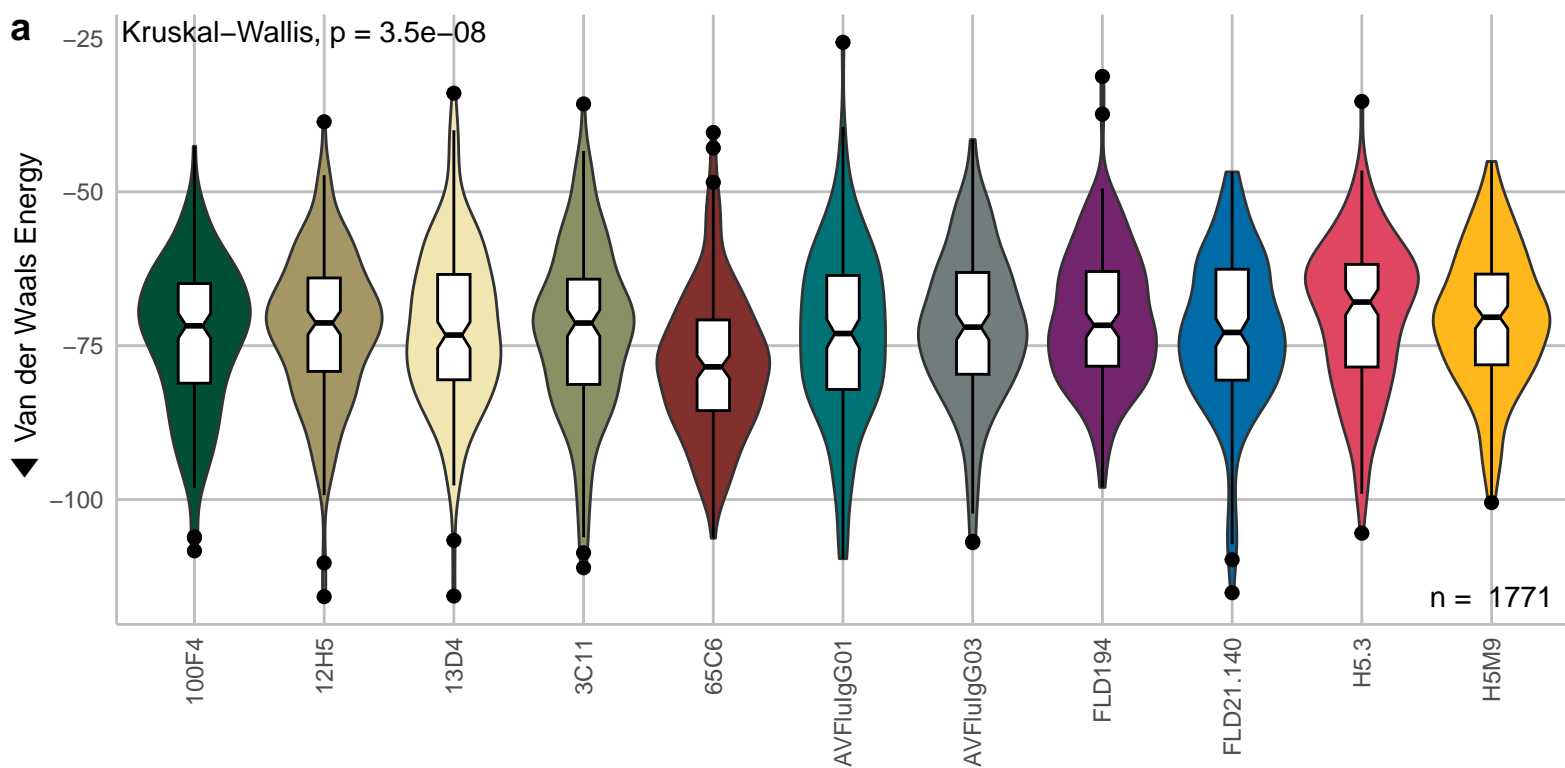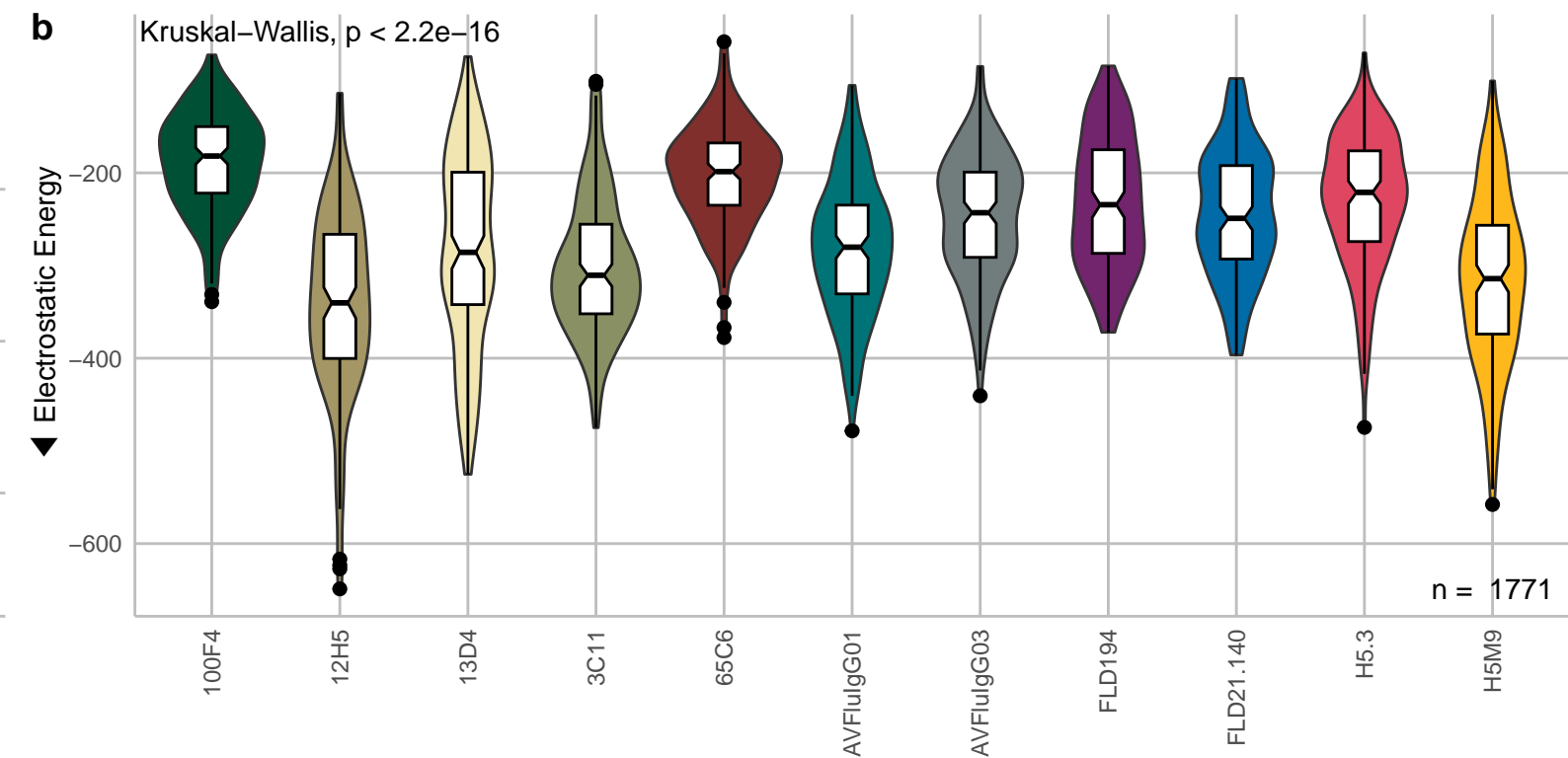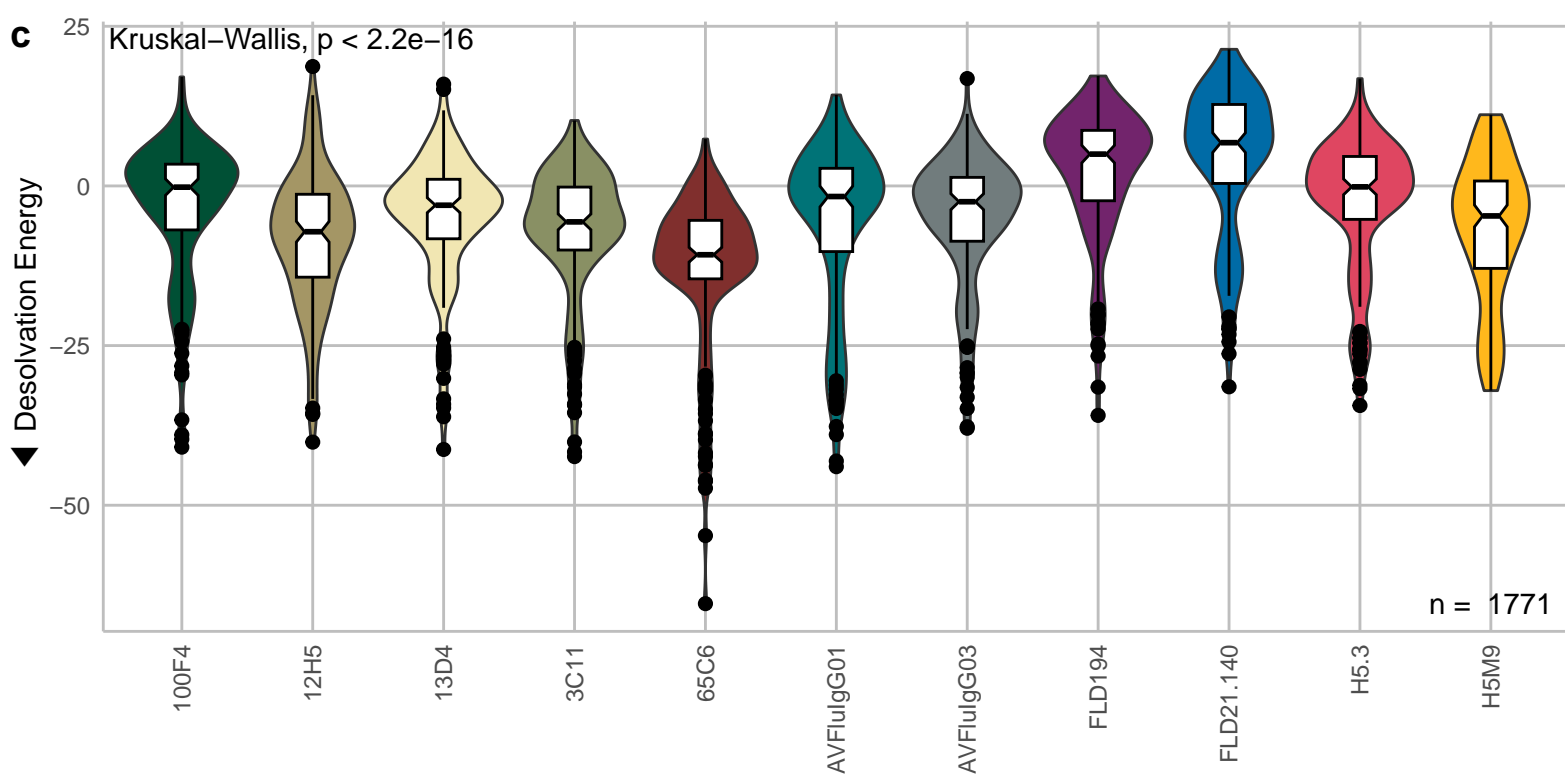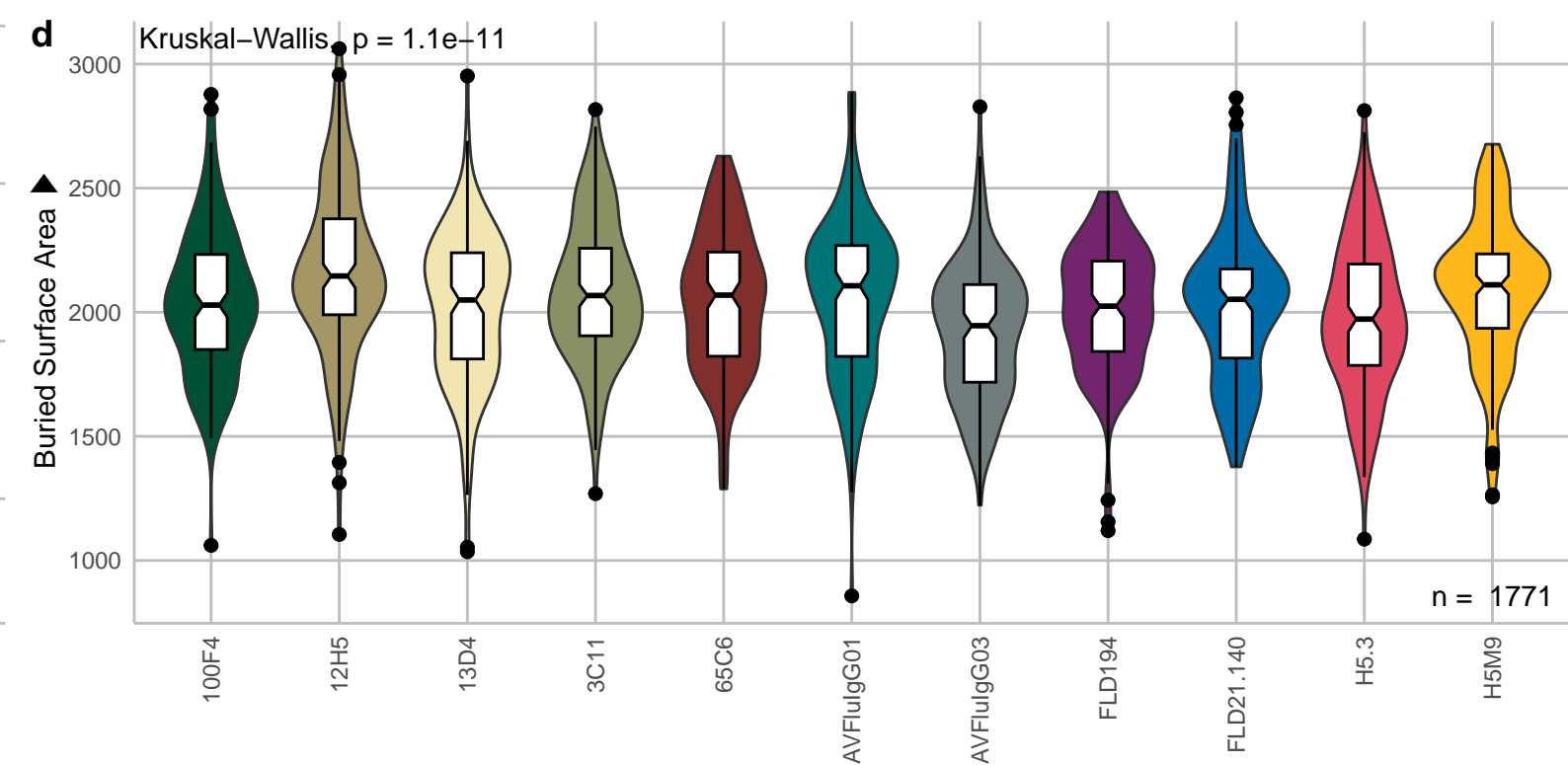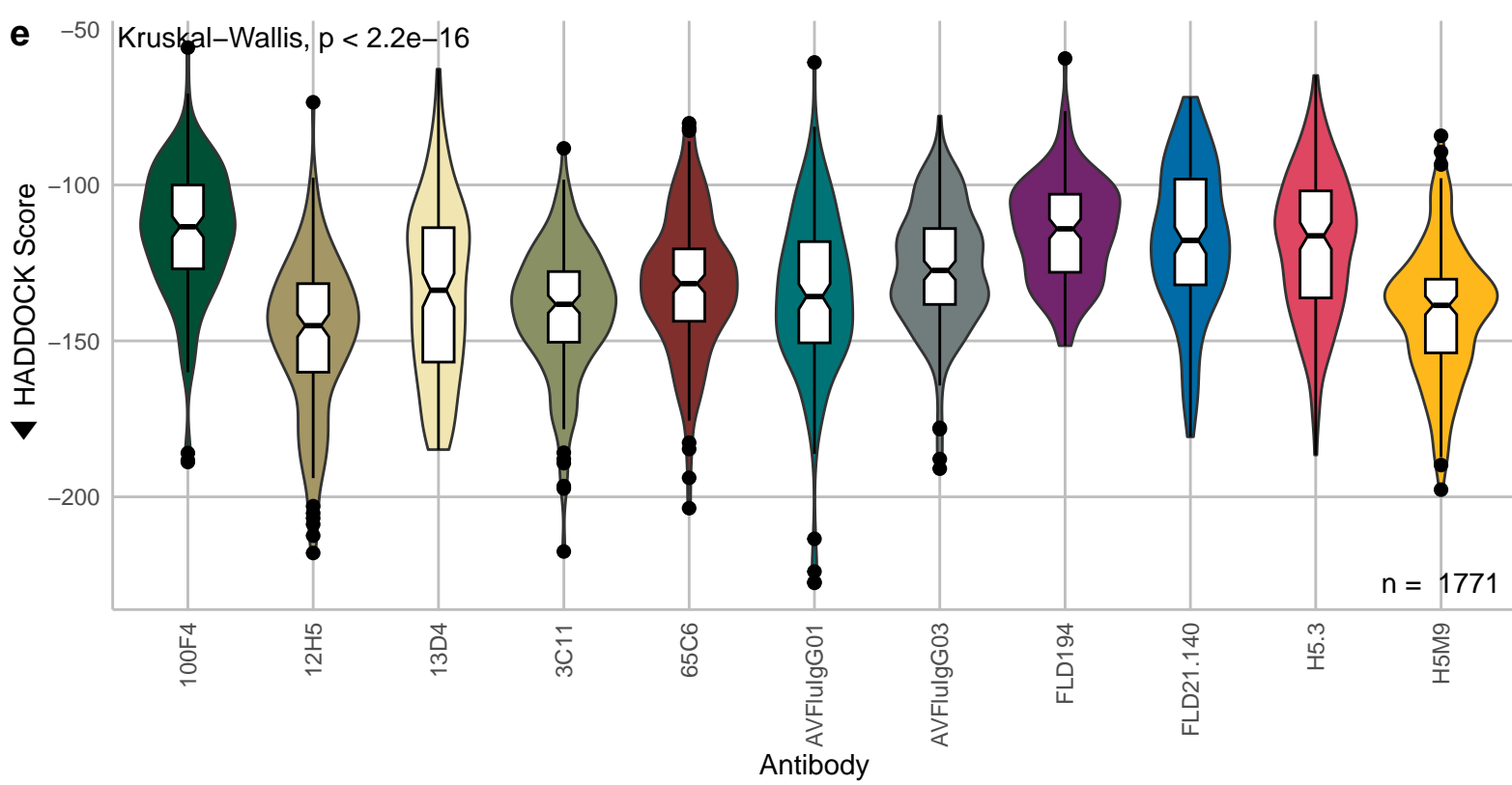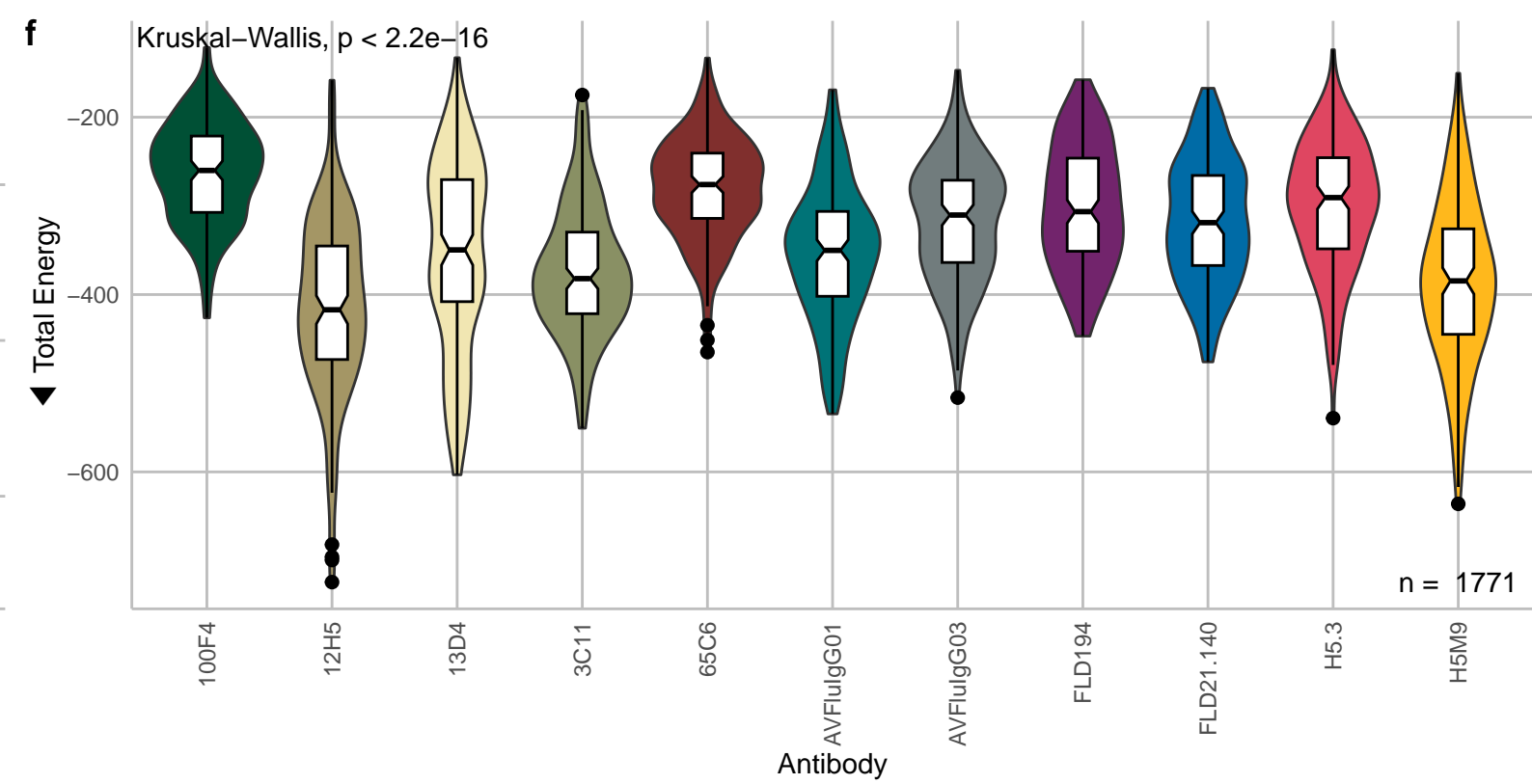

Supplement: Boxplots_by_antibody_grid [file mmc4.pdf]

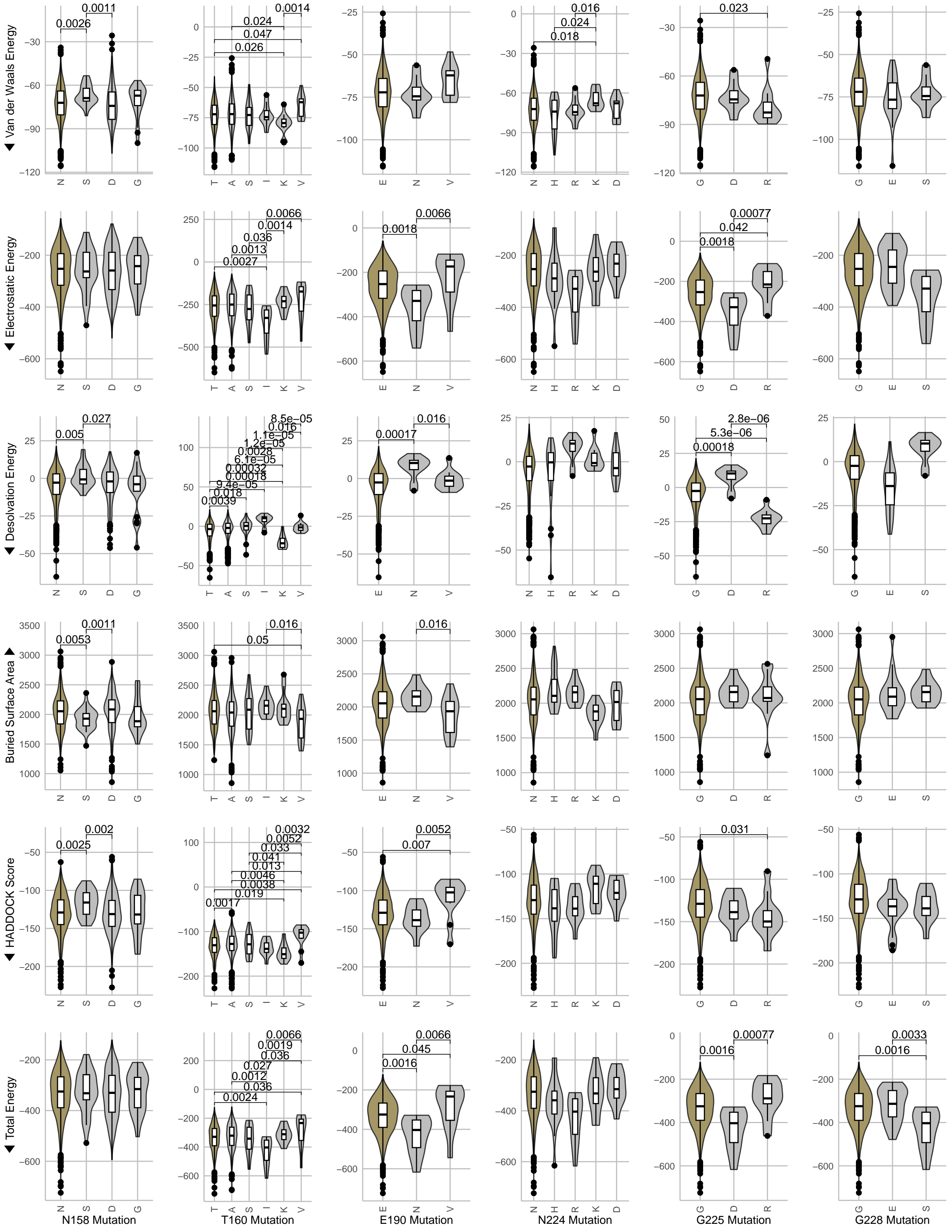

Supplement: Boxplots_by_mutation_metric_grid_vdw [file mmc5.pdf]
